# Immunization Against Inhibin Promotes Fertility in Cattle: A Meta-Analysis and Quality Assessment

**DOI:** 10.3389/fvets.2021.687923

**Published:** 2021-09-21

**Authors:** LingLi Ma, Zhuo Li, ZhongRen Ma, JianBo Ma, Fei Zhao

**Affiliations:** ^1^Key Laboratory of Biotechnology and Bioengineering of State Ethnic Affairs Commission, Biomedical Research Center, Northwest Minzu University, Lanzhou, China; ^2^College of Life Science and Engineering, Northwest Minzu University, Lanzhou, China; ^3^Key Laboratory of Environmental Ecology and Population Health in Northwest Minority Areas, Medicine of Northwest Minzu University, Lanzhou, China

**Keywords:** immunization against inhibin, cattle, fertility, meta-analysis, quality assessment

## Abstract

Superovulation and embryo transfer techniques are important methods in cattle breeding. Combined with traditional superovulation protocols, immunization against inhibin can further improve follicular development and embryo yield. The aim of this study is to determine the efficacy of immunization against inhibin in improving the fertility of cattle through meta-analysis and to provide better clinical veterinary practice guidance. Three English databases (PubMed, EMBASE, Web of Science) were searched for research articles of immunizations against inhibin influence on cattle fertility. Literature screening, data extraction, and meta-analysis were conducted in accordance with Preferred Reporting Items for Systematic Reviews and Meta-Analyses (PRISMA) guidelines. In addition, the Systematic Review Center for Laboratory animal Experimentation (SYRCLE) risk-of-bias (RoB) tool was used to assess the risk of bias of the included animal studies. Potentially relevant studies (317) were identified, and finally 14 eligible studies (all in English) were included. The results of meta-analysis revealed that immunization against inhibin has significant effects on improving the number of ovulations [mean difference (MD) = 0.44, 95% confidence interval (CI) = (0.31, 0.56)], embryos and unfertilized ova [MD = 4.51, 95% CI = (2.28, 6.74)], follicles of the three size categories, the incidence of multiple ovulations [OR = 22.50, 95% CI = (8.13, 62.27)], and the conception rate [OR = 2.36, 95% CI = (1.26, 4.40)]. Moreover, it improved the production of embryos [grades 1 embryos: MD = 3.84, (3.54, 4.15); grade 2 embryos: MD = −0.73, (−0.89, −0.57); grade 3 embryos: MD = −0.50, (−0.75, −0.25); degenerated embryos: MD = 1.16, (−0.51, 2.82); transferable embryos: MD = 2.67, (2.03, 3.31)] and the number of corpora lutea [MD = 1.25, 95% CI = (0.79, 1.71)]. In the above indicators, the differences between the two groups were statistically significant (all *p* < 0.0001). Additionally, according to the quality evaluation results, the risk of bias in the included studies is relatively high. The quality evaluation of the results of the included studies showed that the risk of bias mainly concentrated in the selective, performance, detection, and reporting of bias aspects.

## Introduction

Nowadays, multiple ovulation and embryo transfer techniques are increasingly used in bovine breeding (1). Now, most superovulation (SOV) treatment protocols used are based on the use of pituitary extracts that contain follicle-stimulating hormones (FSH), usually administered twice a day in a decreasing dose for 4 days (2). However, embryos are not very efficient to produce (3). During superovulation, the developing follicle produces an excess of inhibins. Inhibins are dimeric protein hormones synthesized by follicular granulosa cells as α plus βA/βB subunits (encoded by INHA, INHBA, and INHBB, respectively), which were first discovered in 1923 (4–6). A high concentration of inhibins may reduce oocyte quality and embryo development by affecting follicle development (7). Many studies have proved that the biological activity of endogenous inhibins is neutralized by active immunization against inhibins and their subunits as well as elimination of their source, resulting in increased pituitary secretion or plasma concentration of follicle-stimulating hormones, thereby stimulating additional or new follicle growth (8–10). In recent years, the most common method for immunization against inhibins is to use inhibin recombinant DNA vaccines, and many studies have demonstrated that inhibin recombinant DNA vaccines can improve reproductive function in other animals such as rats, mice, hens, and sheep (11–14). Some studies have demonstrated that immunization against inhibins can increase the number of ovulations and improve fertility in dairy cows *in vivo* and *in vitro* (15–17). However, in these studies, the material and method for the preparation of inhibin were different, and the intervention procedures were varied. Moreover, quality assessment of these animal studies reporting the fertility effect of immunization against inhibin is lacking. The objective of this study was to perform a meta-analysis to evaluate the effectiveness of immunization against inhibins in improving reproductive function in cattle.

## Materials and Methods

### Search Strategies

The following databases were searched: PubMed, EMBASE, and Web of Science. The retrieval time for all databases was from the start of the database to January 01, 2021. The keywords we searched by MeSH search and “Title/Abstract” were limited by the search method, which are as follows: (i) (Immunizations OR Immunological OR Immunization OR Immunologic) AND (Inhibin OR Inhibins OR Inhibin-F); (ii) Reproductive OR Fertility OR Fecundability OR Fecundity OR Superovulation; and (iii) Cattle OR bos indicus OR zebu OR zebus OR bos taurus OR domestic cow OR domestic cows OR bos grunniens OR yak OR yaks. The final search strategy was selected as “(i) AND (ii) AND (iii)”. The search was not limited by language. In addition, reference track and Google Scholar (www.scholar.google.com.cn) search were used in a supplementary search.

### Study Selection Criteria

According to the Preferred Reporting Items for Systematic Reviews and Meta-Analyses (PRISMA) guidelines (18), the PICO (Problem/Patient/Population, Intervention/Indicator, Comparison, Outcome) principle was obeyed. The inclusion criteria were prespecified as follows: (i) problem: cows with normal reproductive function; (ii) intervention: treatment with immunization against inhibins, not limited to whether to use other ovulation-stimulating drugs or other treatment options such as superovulation in combination; (iii) comparison: there were control groups in the study, and the control group was not treated with immunization against inhibin; (iv) outcome: fertility, such as ovulation number, number of embryos and unfertilized ova, and numbers of various grades of embryos; and (v) study design: controlled *in vivo* experiments. The exclusion criteria were selected as follows: (i) cows with other diseases; (ii) case report, review article, and letter; and (iii) unreliable or incomplete data.

### Data Extraction

The detailed information of the included studies was collected independently by two authors (Lingli Ma and Zhuo Li) and included (i) the name of the first author and year of publication; (ii) features of the animal model (species, strain, age, weight); (iii) information on the treatment/control group, including fraction of inhibins, amino acid sequence, control drug, sample size, dosage, method of administration, superovulation (intervention of hormone and time of administration), and duration of treatment; and (iv) outcome indicators (the number of ovulations, embryos, and unfertilized ova, follicles of the three size categories, various grades of embryos, degenerated embryos, transferable embryos, corpora lutea, incidence of multiple ovulations, and conception rate). If there are multiple data of the same intervention in experimental groups in a study, each group will be treated as independent data. In addition, some data were only displayed on graphics. In these cases, the corresponding authors were contacted to obtain further information. If there was no response, the data in the graph were measured by the digital ruler software (GetData Graph Digitizer, version 2.25, Russia).

### Quality Evaluation of Included Studies

The risk-of-bias (RoB) tool by the SYstematic Review Center for Laboratory animal Experimentation (SYRCLE) is an adapted version of the Cochrane RoB tool. It is a special tool that contains 10 entries (related to selection, performance, detection, attrition, reporting biases, and other biases) for evaluating the risk of animal experiment bias (19). In this study, we used this tool to assess the risk of bias in animal studies.

### Statistical Analysis

R software (version R x 64 3.4.2, The Cochrane Collaboration, Oxford, United Kingdom) was used for data analysis, and the mean difference (MD) with 95% confidence interval (CI) for continuous variables and odds ratio (OR) with 95% CI for dichotomous variables were calculated. Heterogeneity was assessed by the *I*^2^ and Π^2^ statistical tests. *I*^2^ > 50% was considered to display higher heterogeneity. When higher heterogeneity was obtained, a random-effect model was adopted. Otherwise, a fixed-effect model was used. The sources of heterogeneity were identified and subgroup analysis was performed according to the estrous cycle, inhibin components, and SOV treatment protocols. *p* < 0.05 was considered statistically significant.

## Results

### Literature Screening

**Figure 1:**
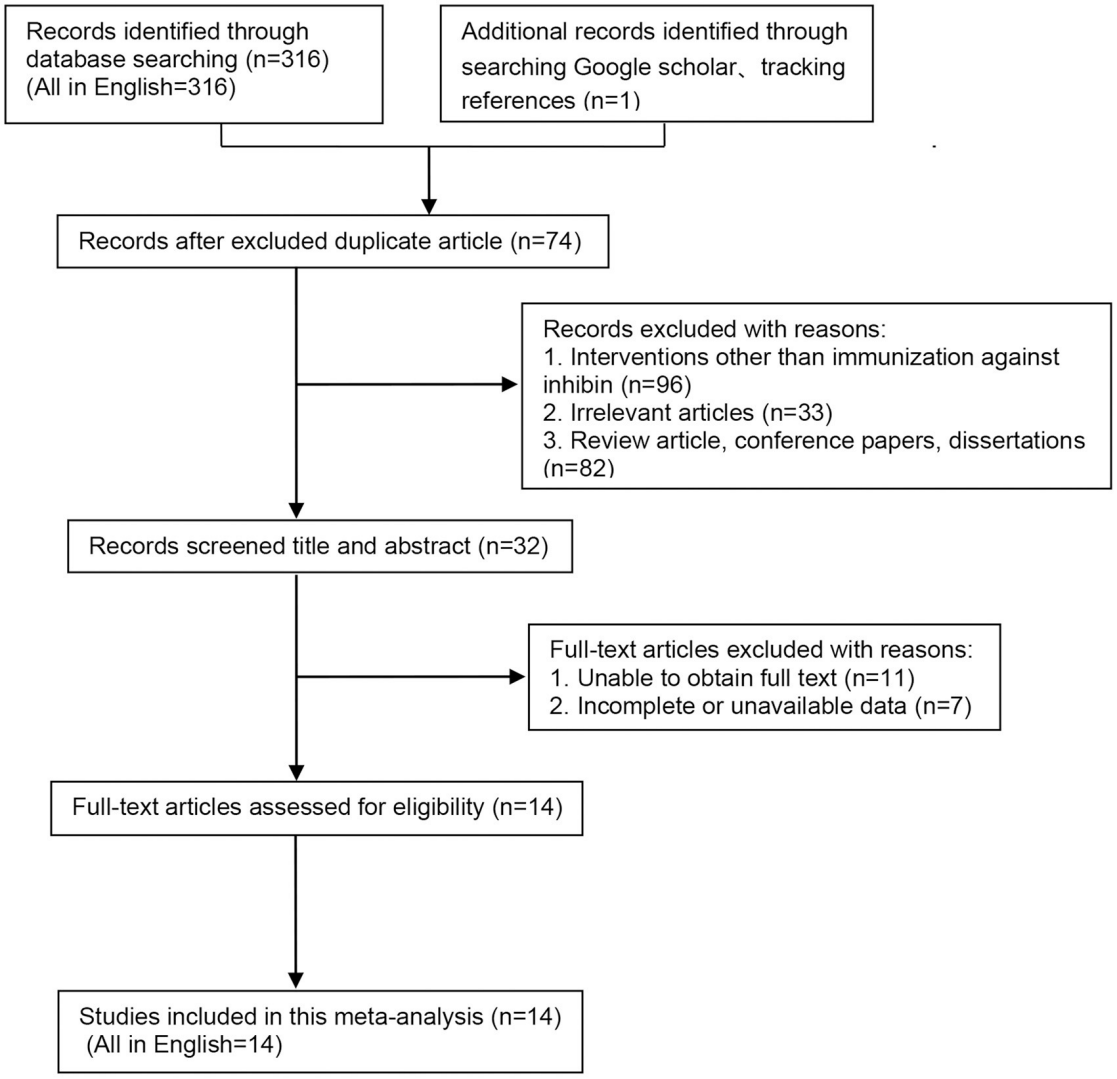
Literature screening flow chart.

Three hundred seventeen potentially relevant studies were identified through database searching (PubMed, *n* = 166; EMBASE, *n* = 18; Web of Science, *n* = 132), and supplementary searching (*n* = 1), of which 74 were duplicated. After screening, 229 studies were excluded as they did not meet the inclusion criteria and exclusion criteria. Finally, as shown in Figure 1, 14 eligible studies (all in English) were selected (5, 16, 20–31).

### Characteristics of the Included Studies

Out of the 14 studies, three (21.43%) corresponded to *in vitro*/*in vivo* research. Regarding the animal, six studies (42.86%) used cow, and six studies (42.86%) used cattle, out of which two studies (14.29%) adopted buffalo. Moreover, eight studies (57.14%) used heifer which are often used in studies on fertility function, the age range was 4–19 months, and five studies (35.71%) used 3–10-year-old cattle. Regarding inhibin, the amino acid sequence and part of the fusion protein were different, but their components were all inhibin alpha subunits. The components of inhibin mainly included bovine inhibin α subunit (BI-α) (five studies, 35.71%), porcine inhibin α subunit (PI-α) (seven studies, 50.00%), and ovine inhibin α subunit (OI-α) (one study, 7.14%). Nine studies (64.29%) reported on vaccine preparation methods, and most of them used DNA recombination methods to produce inhibin alpha subunits in E. coli as immunogens. The outcomes related to fertility mainly involved the following terms: number of ovulations, embryos, and unfertilized ova; follicles of the three size categories; incidence of multiple ovulations; conception rate; the quality of embryos (such as numbers of grade one, grade two, grade three, degenerated, and transferable embryos); and number of corpora lutea. The overall characteristics of the included studies are shown in Table 1.

**Table 1:** Characteristicsof the 12 included studies.

**Study**	**Study design**	**Animal**				**Inhibin vaccine**					**Outcome**
		**Animal species**	**Animal strain**	**Age (months)**	**Weight (kg)**	**Component**	**Fusion protein**	**Amino acid sequence**	**Vector**	**Method**	
Liu et al., (20)	*in vitro*/ *in vivo*	Cow	Holstein	14	NR	PI-α	NR	N-terminal (His)^6^ tag	E. coli	Recombinant DNA	①④⑤⑥
Li et al., (21)	*in vitro*/ *in vivo*	Buffalo	Murrah buffalo	4–5 years	NR	PI-α	NR	N-terminal (His)^6^ tag	E. coli	Recombinant DNA	①⑤⑥⑧
Mei et al., (22)	*in vivo*	Cow	Holstein	16–17	NR	PI-α	NR	N-terminal (His)^6^ tag	E. coli	Recombinant DNA	①④⑤⑥
Li et al., (23)	*in vivo*	Cow	Holstein	14	NR	PI-α	NR	N-terminal (His)^6^ tag	E. coli	Recombinant DNA	①④⑤⑥
Glencross et al., (24)	*in vivo*	Cow	British Friesian	19	413–433	BI-α	Ovalbumin	bI-α-(l-29)Tyr^30^	NR	NR	②
Glencross et al., (25)	*in vivo*	Cow	British Friesian	4	123–143	BI-α	Ovalbumin	bI-α-(l-29)Tyr^30^	NR	NR	③⑦
Takedomi et al., (26)	*in vivo*	Cattle	Japanese black	3–10 years	NR	PI-α	RSA	1–26 (numbering from N-terminal end)	NR	NR	①⑥⑦
Morris et al., (27)	*in vivo*	Cattle	Hereford cross	18	352–368	BI-α	HAS or conalbumin	bI-α_43_-Tyr^6^Gly^7^(8-20)Gly^21^Cys^22^bI-α_43_-Tyr^I52^(153–167)Cys^168^	NR	Recombinant DNA	②③⑦
Morris et al., (28)	*in vivo*	Cattle	Hereford cross	18	360–374	BI-α	HAS or conalbumin	bI-α-Tyr^16^Gly^17^(18–30)bI-α-(63–72)Gly^73^Tyr^74^bI-α-Cys^105^Gly^106^(107-122)	NR	Recombinant DNA	②③⑦
Scanlo et al., (5)	*in vivo*	Cattle	Crossbred beef	14–18	NR	BI-α	HAG	bI-α-1–26 Gly-Tyr	NR	NR	③
Akagi et al., (29)	*in vivo*	Cattle	Japanese black and Japanese brown	5–9 years	455–510	OI-α	NR	NR	E. coli	Recombinant DNA	⑦⑧
Konishi et al., (30)	*in vitro*/ *in vivo*	Cattle	Japanese Black	5–9 years	455–510	PI-α	RSA	1–26 (numbering from N-terminal end)	NR	NR	⑧
Guo et al., (16)	*in vivo*	Cow	Holstein	5–8 years	NR	PI-α	NR	N-terminal (His)^6^ tag	E. coli	Recombinant DNA	⑨
Liu et al., (31)	*in vivo*	Buffalo	Buffalo	NR	NR	I-α	NR	Inhibin α (1–32)	Choleraesuis C500 strain	Recombinant DNA	⑨②

*NR, not reported; BI-α, bovine inhibin α subunit; PI-α, porcine inhibin α subunit; OI-α, ovine inhibin α subunit; I-α, inhibin α subunit; RSA, rabbit serum albumin; HAS, human serum albumin; HAG, human α globulins. ①, Number of embryos and unfertilized ova; ②, ovulation number; ③, incidence of multiple ovulations; ④, numbers of various grades of embryos; ⑤, numbers of degenerated embryos; ⑥, numbers of transferable embryos; ⑦, number of corpora lutea; ⑧, number of follicles of the three size categories; ⑨, conception rate*.

### Characteristics of the Treatment

Table 2 shows in detail the characteristics of intervention. In terms of specific details of the intervention, six studies (42.86%) used saline as control treatment, six studies (42.86%) used fusion protein as control treatment, one study (7.14%) used marcol, and one study (7.14%) used phosphate-buffered saline. The sample size varied from 10 to 84 animals, and the experimental and control groups were consistent in sample size in most studies (eight studies, 57.14%). However, the frequency of treatment was inconsistent among the included studies. The time of primary immunization was 1 day after the experimental start date in most studies (eight studies, 57.14%), but the timing and frequency of the booster varied. All the routes of treatment immunization were injection. Only nine studies (64.29%) mentioned the exact location of the injection, but the injection site was not the same. The number of immunizations was mostly from three (five studies 35.71%) to four (five studies 35.71%), except for two studies which were not mentioned. It is worth noting that the actual dosage of inhibin for the second immunization was half that of the primary immunization in eight studies (57.14%). Twelve studies (85.71%) used SOV treatment protocols at the same time, of which five studies (35.71%) used FSH in SOV treatment protocols, and the other seven studies (50.00%) used other hormones such as prostaglandin in SOV treatment protocols. Two studies (14.29%) did not report whether the SOV has been used. The observation time varied (from 50 to 300 days), except for two studies which were not mentioned.

**Table 2:** Characteristics of inhibin treatment.

**Study**	***T***	***C***	***n* (T)**	***n* (C)**	**Primary immunization**			**Consecutive booster injections**			**Injection site**	**Immune number**	**Superovulation**		**Follow time(day)**
					**Intervention time (day)**	**Concentrations(mg/ml)**	**Dosage (ml)**	**Interventiontime(day)**	**Concentrations (mg/ml)**	**Dosage (ml)**			**Hormone**	**Interventiontime for hormone(day)**	
Liu et al., (20)	PI-α + SOV	Saline + SOV	7	7	1	1	1	28, 56, 88	1	1	NR	4	P + FSH + CLO	62, 94	110
Li et al., (21)	PI-α + SOV	Saline + SOV	8	7	1	1	1	28, 56	0.5	1	NR	3	P + FSH + CLO	68	100
Mei et al., (22)	PI-α + SOV	Saline + SOV	8	10	1	1	1	28, 56	0.5	1	NR	3	P + FSH + CLO	66	90
Li et al., (23)	PI-α + SOV	Saline + SOV	10	8	1	1	1	28, 56	0.5	1	NR	3	P + FSH + CLO	66	100
Glencross et al., (24)	BI-α + SOV	Ovalbumin + SOV	6	6	NR	NR	1mg	Intervals of 1–2 months	NR	0.5 mg	NR	NR	CLO	−28, −14, 0, 14, 28	NR
Glencross et al., (25)	BI-α	Ovalbumin	6	6	NR	NR	1 mg	Intervals of 1–2 months	NR	0.5 mg	Flank	NR	NR	NR	NR
Takedomi et al., (26)	PI-α + SOV	RSA + SOV	14	12	1	NR	1 mg	35, 70	NR	1	Subcutaneously	3	P + E + pFSH + PG	After immunizations	NA
Morris et al., (27)	BI-α + SOV	HAS + SOV	5	5	1	1.5	2	77, 154, 231	0.75	2	Brisket	4	P + PG	82, 159, 236	300
Morris et al., (28)	BI-α + SOV	HAS + SOV	5	5	1	1.5	2	77, 154, 231	0.75	2	Brisket	4	P + PG	82, 159, 236	280
Scanlo et al., (5)	BI-α + SOV	Saline + SOV	8	7	0	NR	1 mg	33, 66, 209	NR	NR	Brisket	4	PG	96, 219, 231	244
Akagi et al., (29)	OI-α + SOV	Marcol + SOV	7	7	9	0.125	1	27, 45, 63	0.125	1	Intramuscularly	3	CLO	63	72
Konishi et al., (30)	PI-α	RSA	6	6	0	NR	1 mg	42, 70, 98	NR	0.5 mg	Neck or shoulder	4	NR	NR	140
Guo et al., (16)	PI-α + SOV	Saline + SOV	11	11	1	1	1	0	0	0	Intramuscularly	1	GnRH + PG	1	50
Liu et al., (31)	I-α + SOV	PBS + SOV	41	43	0	NR	NR	14	NR	NR	Intramuscularly	2	PMSG + PG + GnRH	28	78

*T, treatment; C, control; n, number of cows in the group; NR, not reported; BI-α, bovine inhibin α subunit; PI-α, porcine inhibin α subunit; OI-α, ovine inhibin α subunit; I-α, inhibin α subunit; RSA, rabbit serum albumin; HAS, human serum albumin; HAG, human α globulins; PBS, phosphate-buffered saline; SOV, superovulation; Marcol, the ingredient of placebo (Montanide: marcol adjutant only); P, progesterone; CLO, cloprostenol; PG, prostaglandin; E, estradiol benzoate capsule; FSH, follicle-stimulating hormone; pFSH, porcine follicle-stimulating hormone; GnRH, gonadotropin-releasing hormone; PMSG, pregnant mare serum gonadotropin*.

### Quality Evaluation of the Included Studies

**Figure 2:**
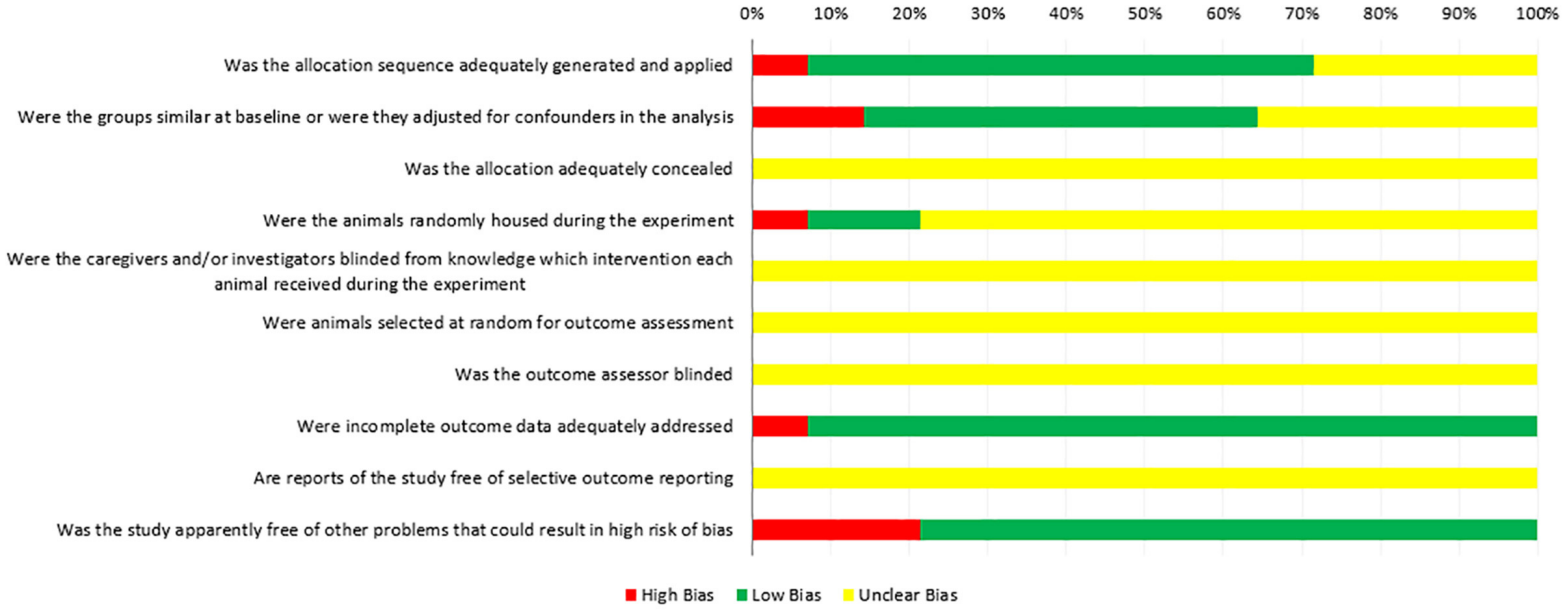
Risk of bias of the included studies by the SYRCLE RoB tool.

Figure 2 shows the quality assessment of the included studies by the SYRCLE RoB tool. All 14 animal experiments were controlled studies; one study (7.14%) used the “age and weight” in sequence generation. Nine studies (64.29%) mentioned “random” in sequence generation, but no study described the specific method of randomization. The bias risk assessment results of “baseline characteristics” in six studies (42.86%) were unclear because the number of animal samples in the experimental and control groups was inconsistent, and one study (7.14%) did not adjust for confounders in the analysis. No study reported the “allocation concealment” clearly, and in the experiment implementation stage, two studies (14.29%) mentioned “random housing to animals,” 11 studies (78.57%) did not mention, one study (7.14%) had a nonrandom placement of animals, and all of the studies did not mention “blinding between investigators and study designers” (100.00%). The same results were obtained by the assessment of “detection bias.” No studies described animals randomly selected for outcome assessment. Thirteen studies (92.86%) reported complete outcome data. All studies clearly reported low bias in “selective outcome reporting.”

### Ovulation Number

**Figure 3:**
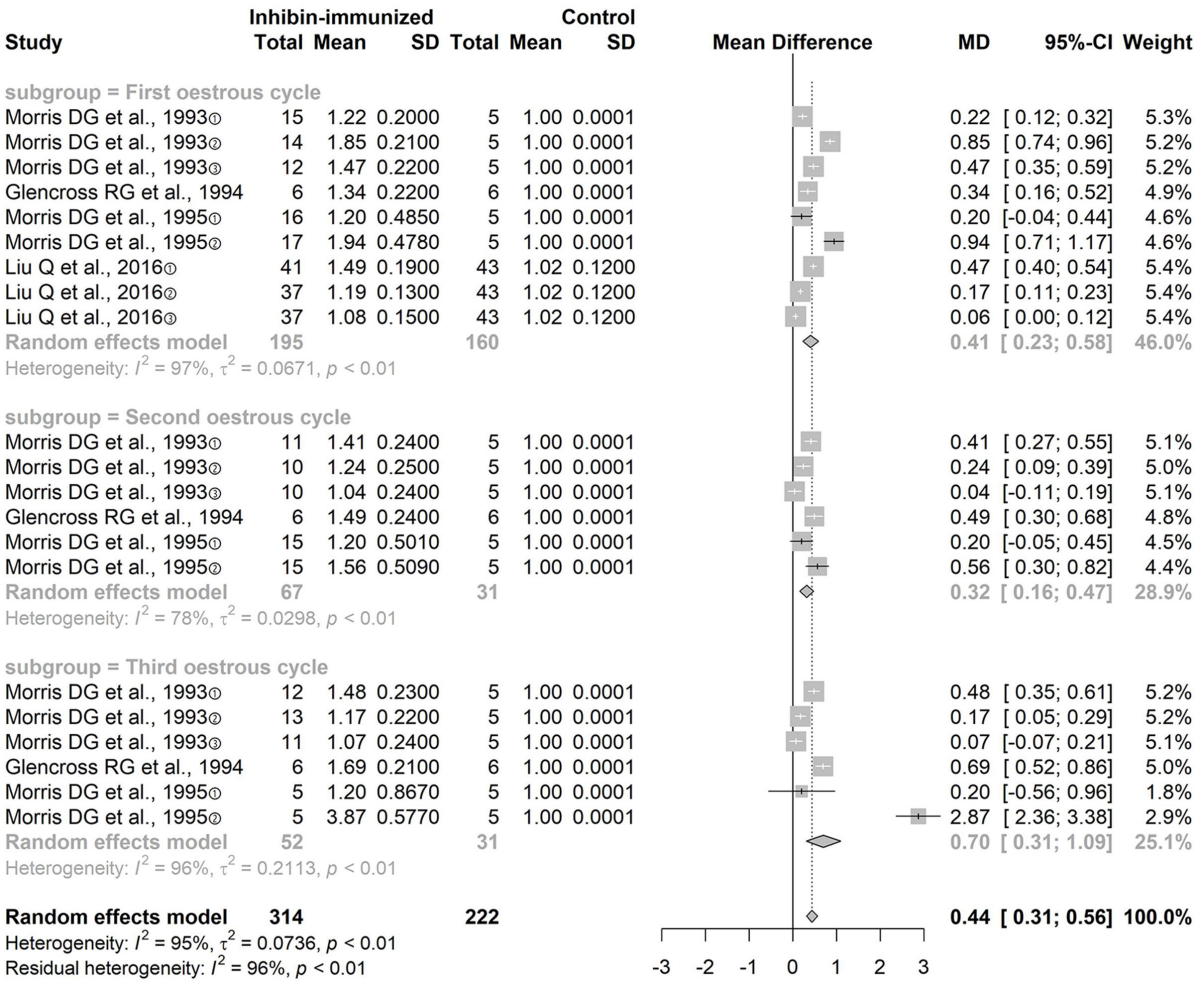
Meta-analysis of ovulation number.

The four included studies all used immunization against inhibin and the combination with SOV treatment protocols that did not contain FSH. We conducted subgroup analyses according to the different estrous cycles: the ovulation number in the first estrous cycle [*n* = 116 MD = 0.41, 95% CI = (0.23, 0.58), *p* < 0.0001; heterogeneity *I*^2^ = 97%], the second estrous cycle [*n* = 116, MD = 0.32, 95% CI = (0.16, 0.47), *p* < 0.0001; heterogeneity *I*^2^ = 78%], and the third estrous cycle [*n* = 116, MD = 0.70, 95% CI = (0.31, 1.09), *p* < 0.0001; heterogeneity *I*^2^ = 96%] (Figure 3). The difference between the two groups was statistically significant. The result of the meta-analysis of immunization against inhibin showed that the number of ovulations in the experimental group was significantly increased during each estrous cycle.

### Number of Embryos and Unfertilized Ova

**Figure 4:**
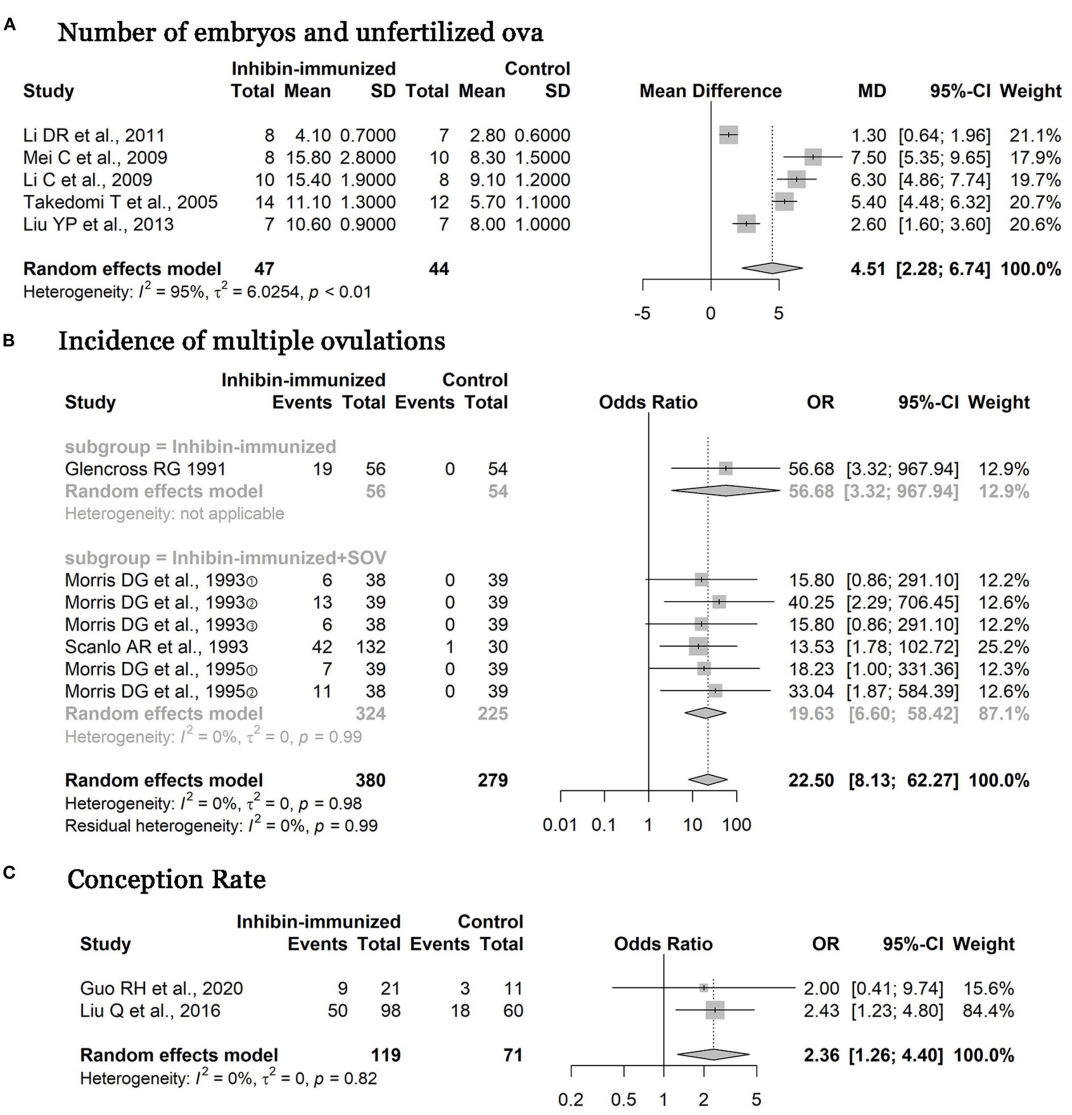
Meta-analysis of number of embryos, unfertilized ova, and incidence of multiple ovulations after treatment and conception rate. SOV, superovulation.

The five studies included in the study all used immunization against inhibin and the combination with the SOV treatment protocols containing FSH at the same time. The meta-analysis showed that immunization against inhibin has significant effects on enhancing the number of embryos and unfertilized ova compared with control [*n* = 91, MD = 4.51, 95% CI = (2.28, 6.74), *p* < 0.0001; heterogeneity *I*^2^ = 95%] (Figure 4A). The difference between the two groups was statistically significant.

### Incidence of Multiple Ovulations

In our assessment, incidence of multiple ovulations means that all estrous cycles multiply ovulations during the period of study in the treatment group compared to the control group. The three studies included in the study used immunization against inhibin and the combination with SOV treatment protocols that did not contain FSH, and one study did not report whether it was combined with SOV treatment protocol. After the subgroup analysis according to hormone types in SOV was carried out, a meta-analysis of four studies showed that immunization against inhibin has apparent effects on enhancing the incidence of multiple ovulations compared with the control treatment [*n* = 47, OR = 22.50, 95% CI = (8.13, 62.27), *p* < 0.0001; heterogeneity *I*^2^ = 0%], and the difference between the two groups was statistically significant (Figure 4B).

### Conception Rate

The two studies included in the study used immunization against inhibin and the combination with SOV treatment protocols that did not contain FSH; the meta-analysis showed that immunization against inhibin has apparent effects on enhancing the conception rate compared with the control treatment [*n* = 106, OR= 2.36, 95% CI = (1.26, 4.40), *p* < 0.0001; heterogeneity *I*^2^ = 0%], and the difference between the two groups was statistically significant (Figure 4C).

### Number of Follicles of the Three Size Categories

**Figure 5:**
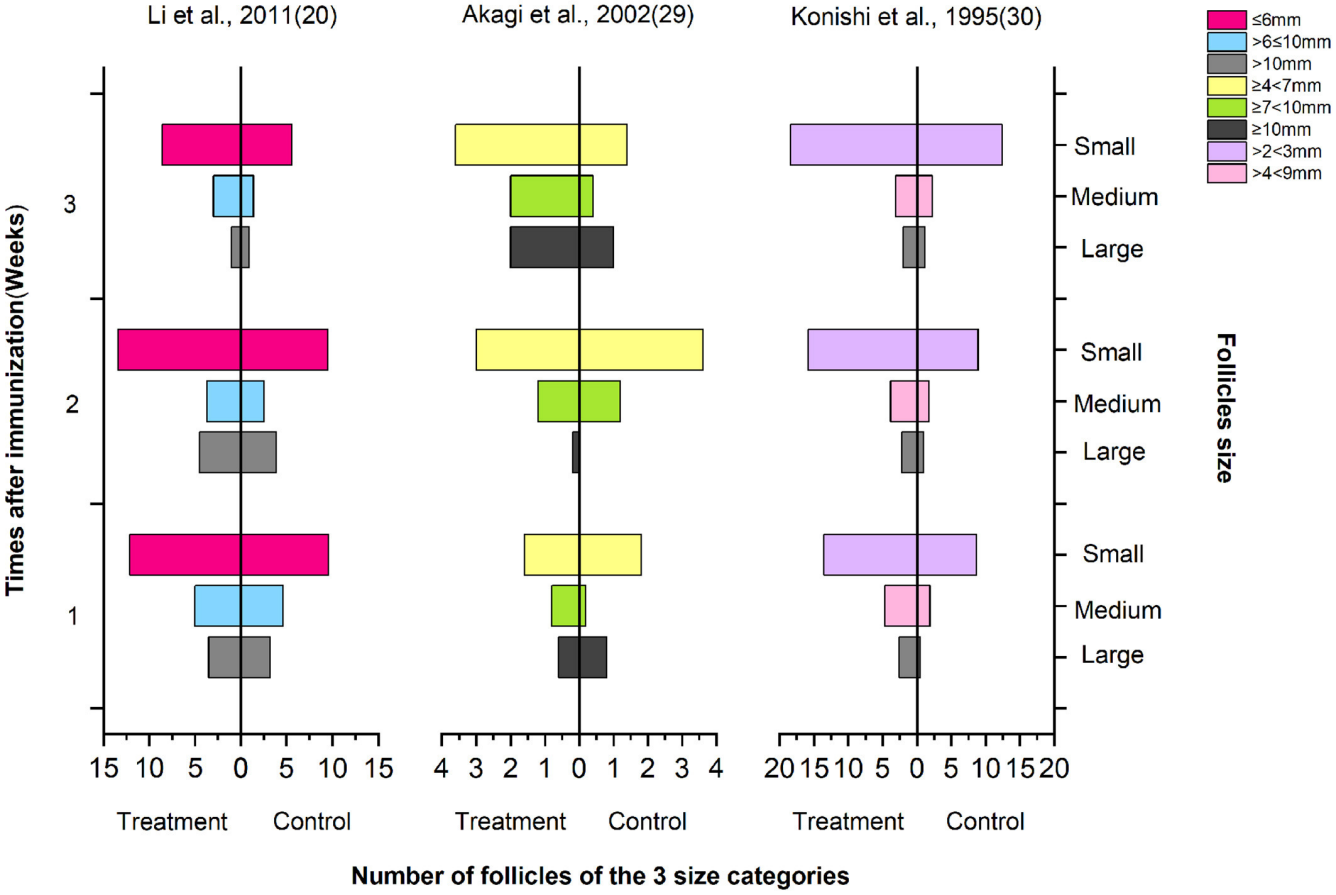
Number of follicles of the three size categories.

As shown in Figure 5, the follicle number of the three size categories, large, medium, and small (according to the authors' report by follicle diameter size in the article), were tested in the first 1, 2, and 3 weeks after immunization. Regardless of the measurement method used, the number of large and medium follicles in the immunized group increased significantly compared with the control treatment. In the figure, Li et al. used immunization against inhibin and the combination with SOV treatment protocol including FSH (21), Akagi et al. used the combined SOV treatment protocol without FSH (29), and Konishi et al. did not report whether it was combined with SOV treatment protocol (30). It shows that compared with other hormones, FSH has a better ovulation stimulation effect in SOV treatment.

### Quality of Embryos

**Figure 6:**
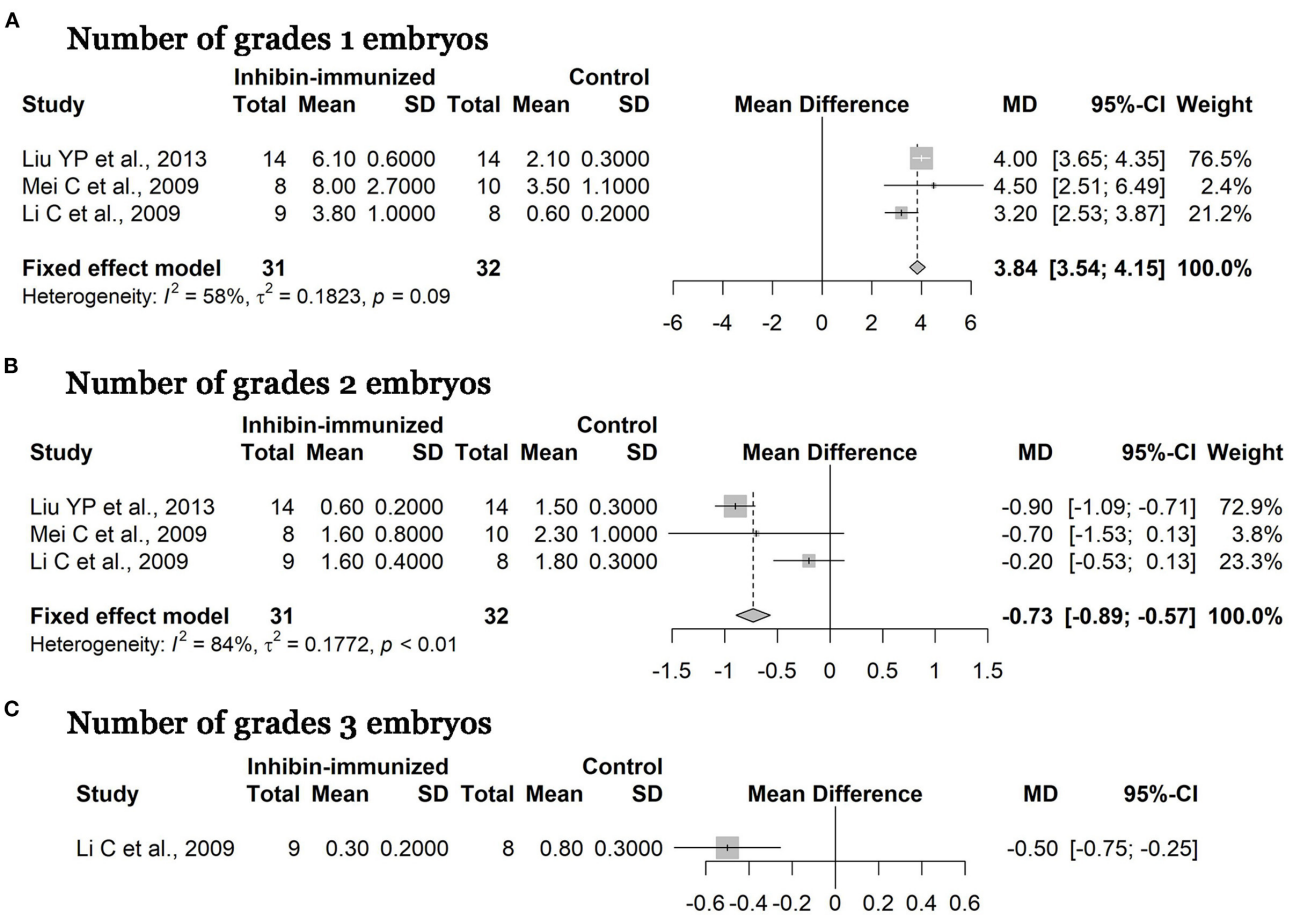
Meta-analysis of numbers of various grades of embryos.

It is worth noting that several studies demonstrated that immunization against inhibin also improved the number of high grades of embryos. After immunized treatment and combination with SOV treatment protocols including FSH, numbers of grade one, grade tow, and grade three embryos (according to the report in the article) in the experimental group all increased compared to the control group. As shown in Figure 6, the MD of numbers of grade one embryos was 3.84 [95% CI = (3.54, 4.15), *p* < 0.0001; heterogeneity *I*^2^ = 58%] (Figure 6A) and that of grade two embryos was −0.73 [95% CI = (−0.89, −0.57), *p* < 0.0001; heterogeneity *I*^2^ = 84%] (Figure 6B). Finally, only one study reported on numbers of grade three embryos, and the MD of the meta-analysis was −0.50 [95% CI = (−0.75, −0.25), *p* < 0.0001] (Figure 6C). The above results suggest that immunization against inhibin is advantageous over the control group regarding its effects on improving the quality of embryos, and the difference between the two groups was statistically significant.

**Figure 7:**
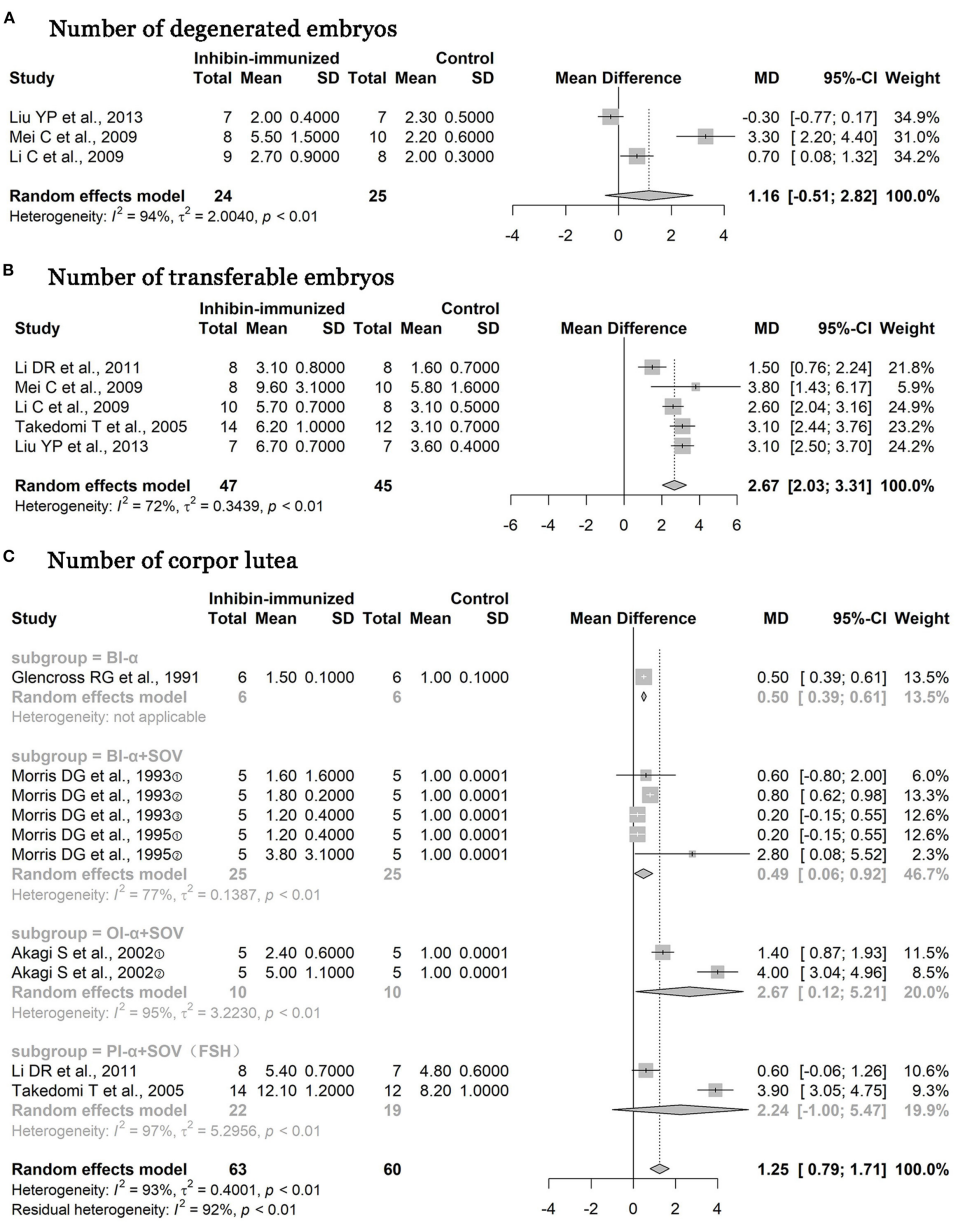
Meta-analysis of numbers of degenerated embryos, transferable embryos, and corpora lutea. BI-α, bovine inhibin α subunit; PI-α, porcine inhibin α subunit; OI-α, ovine inhibin α subunit; SOV, superovulation; FSH, follicle-stimulating hormone.

As shown in Figures 7A,B, studies included in the study all used immunization against inhibin and the combination with the SOV treatment protocols containing FSH at the same time. The numbers of degenerated embryos were reduced [*n* = 49, MD = 1.16, 95% CI = (−0.51, 2.82), *p* < 0.0001; heterogeneity *I*^2^ = 94%] (Figure 7A), but the results showed no statistical significance. In addition, the numbers of transferable embryos increased [*n* = 92, MD = 2.67, 95% CI = (2.03, 3.31), *p* < 0.0001; heterogeneity *I*^2^ = 72%] (Figure 7B), and the difference between the two groups was statistically significant. As shown in Figure 7C, two studies used FSH in SOV treatment protocols, three studies used other hormones in SOV treatment protocols, and one study did not report whether an SOV has been used. After the subgroup analysis according to hormone types was carried out, the number of corpora lutea [*n* = 123, MD = 1.25, 95% CI = (0.79, 1.71), *p* < 0.0001; heterogeneity *I*^2^ = 93%] in the immunized group increased significantly compared with the control treatment, and the difference between the two groups was statistically significant.

## Discussion

As the world's population has increased, the demand for animal products has increased several times. Superovulation and embryo transfer techniques have become important methods in cattle breeding (32–34). Studies have found that one injection of inhibin antigen is equivalent to multiple injections of exogenous gonadotropin in inducing the superovulation response of cattle (7). The combination of inhibin immunity and traditional superovulation schemes makes the superovulation technology of cattle more labor-saving and efficient.

This meta-analysis evaluated the effectiveness of inhibin vaccines in improving reproductive function in cattle. While the search criteria for the meta-analysis included inhibin, the eligible studies, in the end, only included inhibin recombinant DNA as interventions. Most of the interventions in the studies are combined with SOV treatment protocols. In addition, we found that animal strains only included some produce beef cattle and dairy cattle (Holstein, British Friesian, Hereford, Japanese black and brown) as test subjects, except one study which adopted buffalo. The effect of immunization against inhibin on fertility of other commercially cattle such as Brown Swiss dairy cows, Simmental cattle, Jersey cattle, and yaks is therefore unknown. Nevertheless, it is expected to be similar for other commercially relevant cattle.

In this meta-analysis, compared with the control treatment, the numbers of ovulations [MD = 0.44 (0.31, 0.56)], embryos, and unfertilized ova [MD = 4.51, (2.28, 6.74)] were significantly greater in the inhibin-immunized group. The incidence of multiple ovulations [OR = 22.50, (8.13, 62.27)] and the conception rate [OR = 2.36, (1.26, 4.40)] also significantly increased. These results supported the conclusion that the fertility of cattle immunized with inhibin recombinant DNA was significantly improved. The analysis results of numbers of follicles of the three size categories showed that there are also increases in the quality of ovulations, especially in combination with SOV treatment protocols including FSH. In addition, the number of high-quality embryos also significantly improved after immunization against inhibin treatment, including the numbers of various grades of embryos, transferable embryos, and corpora lutea. A *p* = < 0.0001 revealed that the difference between the two groups was statistically significant, except for the results of the numbers of degenerated embryos [MD = 1.16, (−0.51, 2.82), *p* < 0.0001]. Inhibin is a factor secreted by developing ovarian follicles, and it has the effect of inhibiting the secretion of FSH from the pituitary gland and the development of follicles. Studies have shown that immune neutralization of inhibin would reduce its endogenous production, accompanied by increased pituitary activity, increased FSH, and increased follicular growth wave (20, 21). All of these have obvious effects on improving the quality of follicles and embryos produced. The results of this study are also consistent with these reports.

However, it is worth noting that the heterogeneity of the meta-analysis of ovulation number, number of embryos and unfertilized ova, and high grades of embryos, transferable embryos, and corpora lutea was high (>50%) and remained high even after sensitivity analysis and subgroup analysis. This indicates that the source of heterogeneity is clinical. Moreover, we found that 12 studies used the SOV treatment protocols to treat at the same time, so we also took it as one of the factors that have an impact on the experiment results. Next, we enumerate the possible sources of heterogeneity. (i) The strain, species, age, and sample size of the animal used in the included studies were different. For example, some studies used Holstein cow, whereas others used Japanese black cattle. It is known that the biological characteristics of cattle with different ages (heifer/mature) and strains (Holstein/Murrah buffalo/British Friesian/Japanese black/Hereford cross/Japanese brown) differ, which inevitably affects the outcome of the experiments. Especially, the advanced age of animals and their different physiological conditions may produce heterogeneity in meta-analysis. Therefore, the characteristics of each animal and species are also important sources of influencing factors of heterogeneity. (ii) The fractions of the inhibin amino acid sequence and fusion protein used in the included studies were also different, and the types of hormones used in SOV treatment protocols were different. After the subgroup analysis according to inhibin components and hormone types was carried out, the heterogeneity has not been reduced, so the difference in the inhibin amino acid sequence and fusion protein and the difference in hormones used in SOV treatment protocols were also the reasons for the heterogeneity. (iii) The treatment features (such as intervene time, concentration, dosage, injection site, and number of immune) of the experimental and control groups were also not uniform. These will affect the results of the experiment; for example, different injection sites will affect the absorption and effect of the drug (35, 36). (iv) The grading standards of follicles and embryos are inconsistent. These differences directly affected the experimental results. We cannot ignore the impact of inconsistency and non-standardization of animal experiment design and measurement methods on the heterogeneity of results. (v) The number of animal samples in the experimental and control groups was inconsistent. These results demonstrate that careful attention must be paid to experimental design and source of animals; other baseline characteristics of animals and methods of measurement should be kept consistent.

Additionally, we paid attention to the quality evaluation of these studies. In 2014, the Cochrane Collaboration developed an RoB tool to assess the quality of animal experiments to avoid the risk of bias in animal experiments (37). In our study, the quality evaluation of the results of the included studies (Figure 2) showed that the risk of bias in most animal experiments was mainly concentrated in the following aspects. (i) Selective bias: some studies did not report specific random grouping methods in “sequence generation,” even not adjusted for confounder baseline in the analysis. In all these studies, allocation was not adequately concealed. (ii) Performance biases: most of the studies did not report random housing and the caregivers (investigators) were blinded from knowledge on which intervention each animal received during the experiment. (iii) Detection biases: “blinded” was ignored in the detection. No blinding to animal keepers may lead to subjective bias in their expected experimental results. (iv) Reporting bias: in general, negative/no data results are more difficult to publish than positive/data-based results. Improper handling of missing or unsatisfactory data can lead to reporting bias to a large extent. Therefore, it is necessary to encourage the registration of animal experiments and reporting incomplete data problems. In addition, the following factors need to be considered: (i) experimental animals should have consistent baseline characteristics such as species, source, and age, in accordance with reporting specifications. (ii) Specific experimental methods (such as intervention time, concentration, dosage, injection site, and number of immunizations) should be consistent. (iii) This meta-analysis also highlighted the need for consistent methods of measurement. For instance, some studies counted three types of follicles (small, medium, and large), but this size was measured using eight different measures: ≤ 6 mm, >6 ≤ 10, >10, ≥4 <7, ≥7 <10, ≥10, >2 <3, and >4 <9 mm. This difference in measurement methods prevents the veracity of studies on the effects of immunization against inhibin on the fertility function of cattle.

Unavoidably, this study has some limitations. On the one hand, due to the lack of databases specifically for clinical veterinary research, only three English databases were searched, which may lead to selective bias (38). On the other hand, the diversity of measurement standard of follicles and embryos limits our ability to evaluate fertility after immunization against inhibin.

## Conclusion

In this study, for the first time, meta-analysis and quality evaluation methods were used to evaluate the fertility of cattle vaccinated with inhibin. All analyses showed that compared with the control treatment, the immunization against inhibin recombinant DNA produced a significant effect of promoting fertility. Thus, immunization against inhibin makes the superovulation technology of cattle more labor-saving and efficient. The risk of bias in most animal experiments was mainly concentrated in the selectivity, performance, detection, and reporting of bias. We recommend that future research designs focus on these aspects.

Finally, the diversity of measure of follicles and embryos was an important factor affecting the results. It limits the refinement of policymaking for high follicles and embryos. Standardizing definitions and research methods will improve people's ability to understand and manage superovulation.

## Data Availability Statement

The raw data supporting the conclusions of this article will be made available by the authors, without undue reservation. The datasets presented in this study can be found in online repositories. The names of the repository/repositories and accession number(s) can be found in the article/Supplementary Material.

## Author Contributions

LM, ZL, and FZ conceived and designed the study. LM and JM conducted the systematic review of the literature, extracted data and drafted the manuscript. FZ provided statistical analysis support. ZM supervised the study. All authors read and approved the final manuscript.

## Funding

The National Natural Science Foundation (NSFC) of China (81860716). The University Innovation Ability Improvement Project of Gansu Provincial Department of Education (2019B-023). The Fundamental Research Funds for the Central Universities of North west Minzu University (31920190096). The Characteristic discipline of bioengineering construction for the special guide project of the world-class universities and world-class disciplines of Northwest Minzu University (11080306). Northwest Minzu University for Nationalities Double First Class special bioengineering disciplines (10018703).

## Conflict of Interest

The authors declare that the research was conducted in the absence of any commercial or financial relationships that could be construed as a potential conflict of interest.

## Publisher's Note

All claims expressed in this article are solely those of the authors and do not necessarily represent those of their affiliated organizations, or those of the publisher, the editors and the reviewers. Any product that may be evaluated in this article, or claim that may be made by its manufacturer, is not guaranteed or endorsed by the publisher.
