# Supplementary figures and images for: Immunization Against Inhibin Promotes Fertility in Cattle: A Meta-Analysis and Quality Assessment
Source: Front Vet Sci. 2021 Sep 21;8:687923. doi: 10.3389/fvets.2021.687923 (PMC8490720; doi:10.3389/fvets.2021.687923)

Web of Science n=132


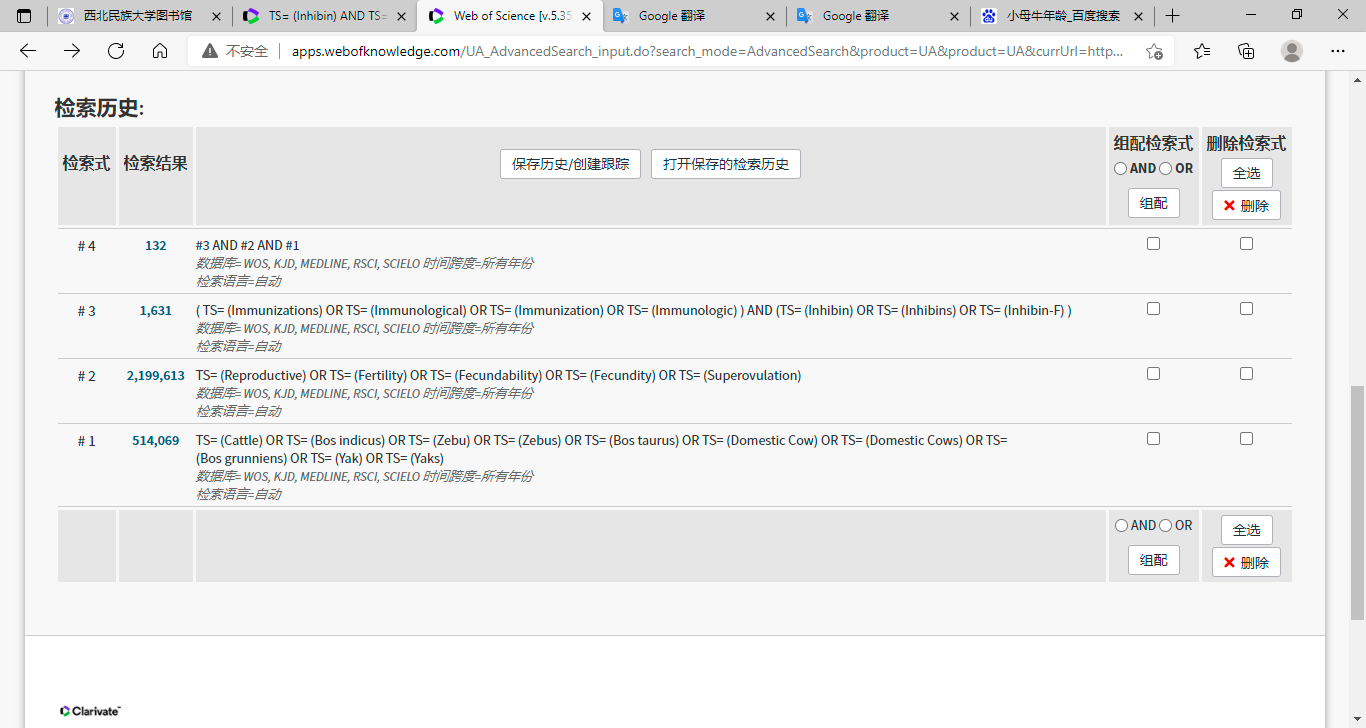


Pubmed n=166


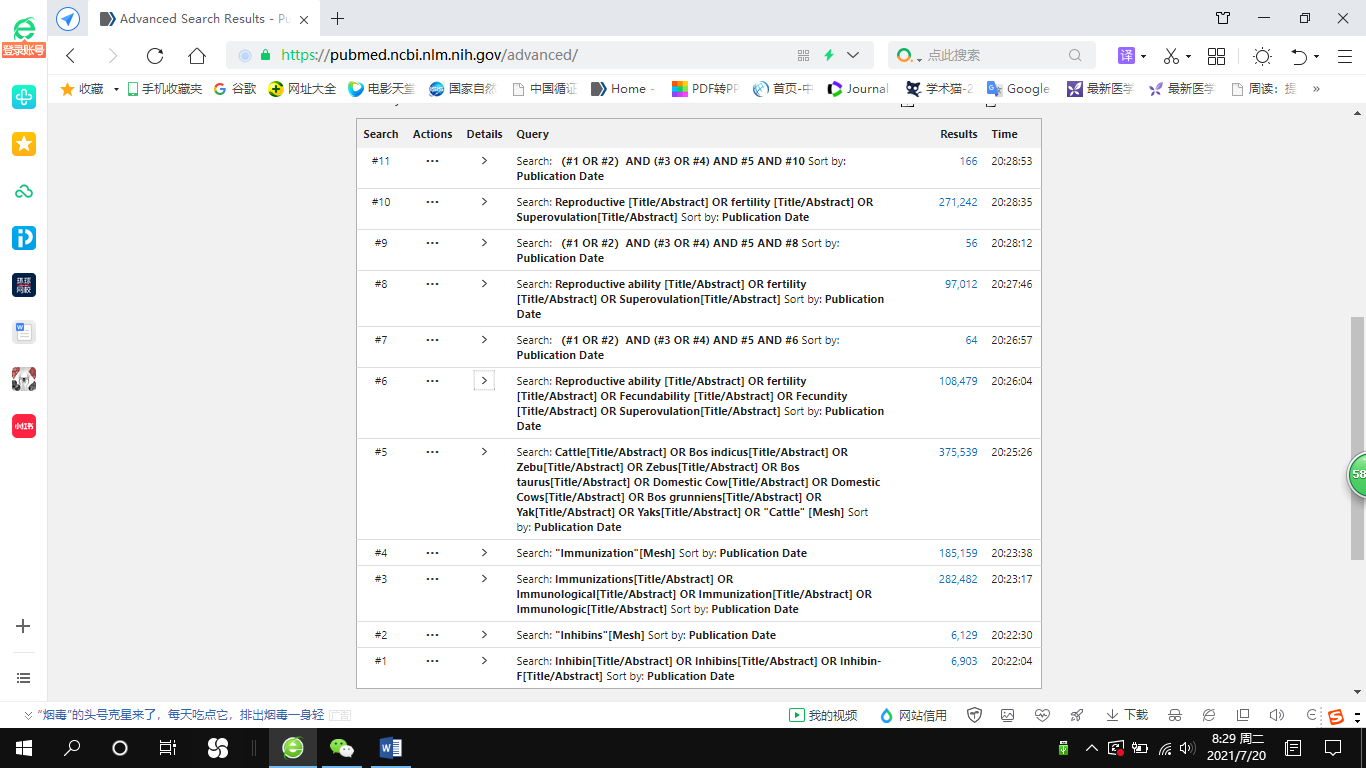


Embase n=18


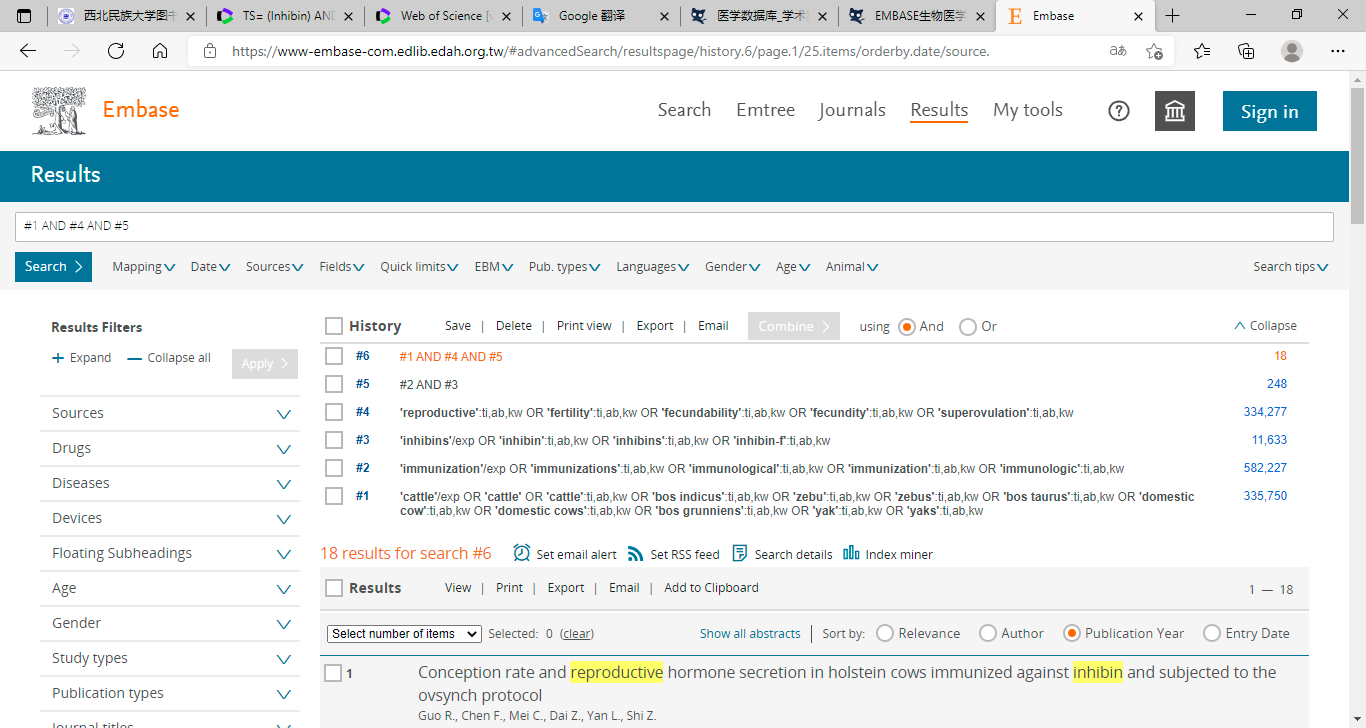

Supplement: Supplementary file 2 [file Data_Sheet_2.docx]
